# Genomic characterisation and antimicrobial resistance of *Klebsiella* species isolated from small stock and environmental sources in South Africa

**DOI:** 10.3389/fcimb.2026.1743913

**Published:** 2026-03-11

**Authors:** Tshepang Motlhaping, Deidré Van Wyk, Oriel Thekisoe, Henriette Van Heerden, Kgaugelo E. Lekota, Tsepo Ramatla

**Affiliations:** 1 Unit for Environmental Sciences and Management, North-West University, Potchefstroom, South Africa; 2 Department of Veterinary Tropical Diseases, Faculty of Veterinary Science, University of Pretoria, Onderstepoort, South Africa; 3 Centre for Applied Food Safety and Biotechnology, Department of Life Sciences, Central University of Technology, Bloemfontein, South Africa

**Keywords:** antibiotic resistance, average nucleotide identity, *Klebsiella* species, virulence genes, whole genome sequencing

## Abstract

*Klebsiella* species pose a significant public health concern due to their association with various infections and the rising levels of antibiotic resistance. This study examined the antibiotic resistance profiles of nine *Klebsiella* species obtained from the culture collection at North-West University’s Potchefstroom campus in South Africa using whole genome sequencing. The average nucleotide identity (ANI), plasmid types, and the identification of antibiotic resistance and virulence genes were performed on *Klebsiella* species. The nine sequenced *Klebsiella* spp. isolates were identified as *K. pneumoniae* (n = 2) originated from sheep faeces, *K. variicola* (n = 2) and *K. michiganensis* (n = 5) isolated from water (river stream). The genomes of *K. variicola* and *K. michiganensis* contained antibiotic resistance genes for fosfomycin (*fosA*), nalidixic acid (*oqxAB*), and β-lactamase (*bla*_LEN24/16_ or *bla*_OXY-1-3_), aminoglycoside (*aph(3’)-Ia_5*), efflux pump [*mdf(A*)] and tetracycline [*tet(34*)]. Furthermore, *K. michiganensis* strains harboured the aminoglycoside *aph(3’)-Ia* gene. *K. pneumoniae* strains KPT2 and KPT4 contained a plasmid AB595 that encodes for tetracycline (*tetA*), sulphonamide (*sul*), and aminoglycoside (*aph(3’)-Ia*). Core virulence genes encoding siderophores (*ent*, *fep*, *iutA*), adhesins (*fim*, *ecp*), and quorum sensing (*luxS*) were present across *Klebsiella* spp. isolates. Additional flagellar-related genes, including those for motor assembly (*fliE, fliF, fliG, fliH, fliI*, and *fliJ*) and motility (*motA* and *motB*), were exclusive in the *K. pneumoniae* strain KPT4, highlighting their crucial role in biofilm formation and survival under antibiotic stress. The identification of ARGs, including *bla*_SHV-194_, *fosA6*, *oqxAB*, and *tet(A)*, in *K. pneumoniae* isolates from sheep further highlights the circulation of antimicrobial resistance determinants in low-resource settings, where surveillance is limited and exposure to untreated animal waste is prevalent. This study provides genomic evidence that rural agricultural environments serve as underappreciated multidrug-resistant and potentially virulent *Klebsiella* species reservoirs and underscore the need for integrated One Health surveillance in low-resource farming communities.

## 1 Introduction

The *Klebsiella* genus belongs to the *Enterobacteriaceae* family, comprising non-motile, Gram-negative, rod-shaped bacteria capable of thriving in aerobic and anaerobic environments (Bengoechea and Sa Pessoa, 2019). They are opportunistic pathogens with a global distribution (Alcántar-Curiel et al., 2018) and are commonly found in the microflora of animals and humans, playing a significant role in intestinal health (Holt et al., 2015). Currently, the *Klebsiella* genus includes many species, such as *K. pneumoniae, K. oxytoca, K. ozaenae, K. rhinoscleromatis, K. terrigena, K. planticola, K. michiganensis, K. variicola*, and *K. ornithinolytica* (Gómez et al., 2021; Palusiak et al., 2025).

Members of this genus typically produce two types of cell surface antigens: lipopolysaccharide (LPS) and capsular polysaccharide (CPS) (Choi et al., 2020). These antigens are utilised for serotyping: the CPS structure determines K-typing, while the O-specific polysaccharide (O-SP) structure of LPS determines O-typing (Podschun and Ullmann, 1998). Antibodies targeting the capsule protect against encapsulated strains (Salazar et al., 2023). Although 12 O-antigen serotypes were initially described, subsequent detailed chemical analyses revealed that four of these shared identical structures. Consequently, only eight distinct O-antigen serotypes are currently recognized (Slater et al., 2024).

*Klebsiella* spp. are known to cause various infections, including nosocomial bronchopneumonia, septicaemia, and urinary tract infections, many of which are resistant to multiple antibiotics (Idomir and Nicoara, 2023; Le et al., 2023). This poses a serious healthcare issue, as *Klebsiella* spp. can evade the immune system and present significant challenges in medical treatment due to rising antibiotic resistance and highly virulent strains (Ballén et al., 2021). They are becoming increasingly resistant to many antibiotics, most recently carbapenems (Mancuso et al., 2023). Several studies indicate that *Klebsiella* strains often show resistance to cephalosporins, levofloxacin, lincomycin, sulfamethoxazole, and ampicillin (Idomir and Nicoara, 2023; Tran et al., 2023). Importantly, *Klebsiella* isolates demonstrate susceptibility to colistin, carbapenems, gentamicin, and ceftiofur (Sivaramakrishnan et al., 2022). The World Health Organization (WHO) has classified *Klebsiella pneumoniae* as a critical pathogen owing to its high resistance to multiple drugs and the urgent need for new treatment options (WHO, 2022). Among *Klebsiella* isolates, resistance due to extended-spectrum *β*-lactamases (ESBL) is most prevalent, followed by ampicillin class C (*ampC*) and carbapenemase production (Garcia-Fierro et al., 2022). In addition, virulence factors such as fimbrial adhesin H (*fimH)*, mannose-resistant *Klebsiella*-like adhesin D (*mrkD*), and enterobactin synthase component B (*entB*) are found in *K. pneumoniae* isolates, contributing to their pathogenicity and increasing the risk of mortality in infected individuals (Mirzaie and Ranjbar, 2021).

Identifying antibiotic resistance and virulence genes in *Klebsiella* spp. is crucial for understanding their pathogenicity and guiding therapeutic approaches. Studies have demonstrated varying antibiotic resistance patterns in *K. pneumoniae* strains, as well as the recurrent presence of carbapenem resistance genes such as *β*-Lactamase *K. pneumoniae* carbapenemase-2 (*bla*_KPC-2_) (Liu et al., 2023; Bonda et al., 2023; Li et al., 2023). The genetic diversity and evolutionary relationships of *Klebsiella* species isolated from animals and water sources can be elucidated through comparative genomics. Whole genome analysis of *Klebsiella* strains from these distinct environments can reveal genetic variants that highlight differences in antibiotic resistance, pathogenic potential, and adaptive processes. This method elucidates potential transmission pathways between aquatic habitats and animals, providing insight into the risks of zoonotic diseases and environmental contamination. Understanding these genetic variations aids in evaluating public health implications and developing targeted strategies for surveillance and managing *Klebsiella* infections in diverse contexts.

*Klebsiella* species are increasingly implicated in both hospital- and community-acquired infections, and their growing resistance to multiple antibiotics, especially in low-resource and under-surveilled environments, poses a critical threat to global health. Despite growing concerns about antimicrobial resistance (AMR), genomic surveillance of *Klebsiella* species in animals and the environment remains critically underexplored, especially in rural South African communities like Matlwang village, Potchefstroom, North West Province. This knowledge gap hampers our ability to trace the genetic drivers of resistance and virulence, particularly in areas where humans, livestock, and water sources interact closely. Without comprehensive whole-genome data, emerging resistant strains remain undetected, posing a silent threat to animals and public health. To address this, our study sought to uncover the genomic profiles of *Klebsiella* spp. circulating in these environments by characterizing their antibiotic resistance genes, virulence factors, and genetic diversity through whole genome sequencing. By filling these knowledge gaps, the study aims to inform targeted interventions, highlight the role of rural environments in AMR dissemination, and contribute to more inclusive and geographically representative AMR databases and strategies.

## 2 Materials and methods

### 2.1 Bacterial isolates and genomic DNA extraction of the *Klebsiella* spp.

The 9 *Klebsiella* spp. isolates, comprising 5 from *K. michiganensis*, 2 from *K. pneumoniae*, and 2 from *K. variicola*, were selected based on the available strains from the Microbiology Biobank at North-West University’s Potchefstroom Campus in South Africa. The original faecal samples were collected directly from the rectum of sheep. In contrast, water samples were collected from river streams using sterile bottles as part of the AMR surveillance research collected from a rural communal farm in Matlwang, Potchefstroom. The original specimens, comprising fresh faecal material from sheep and water samples, were aseptically obtained between February 2022 and April 2022 and transported to the laboratory under cold-chain conditions. Previously stored at -80°C in nutrient broth with 15% sterile glycerol, the isolates were revived by subculturing on Eosin Methylene Blue (EMB) agar and incubating at 37°C for 24 hours.

### 2.2 Identification of the isolates using the SensiTitre system

The SensiTitre system was used in this study to identify Gram-negative bacterial isolates. Bacterial colonies were first cultured on nutrient agar and incubated overnight at 37°C. Isolated colonies were then emulsified in sterile saline (0.85% NaCl) to achieve a turbidity equivalent to a 0.5 McFarland standard, which was confirmed using a densitometer. The prepared bacterial suspension was dispensed into the wells of a Gram-negative SensiTitre identification panel, containing specific biochemical substrates, following the manufacturer’s instructions (Thermo Fisher Scientific Inc., Waltham, MA, USA). The plates were sealed and incubated at 37°C for 18–24 hours under aerobic conditions. After incubation, the results were either visually read or interpreted using an automated reader. Biochemical reactions, such as colour changes and gas production, were recorded and compared against the SensiTitre database using the system’s software or manual reference tables for species identification.

### 2.3 Genomic extraction of the isolates

The genomic DNA was extracted from overnight culture broths of *Klebsiella* spp. isolates using a ZymoBIOMICS DNA extraction kit following the manufacturer’s instructions (Zymo Research Corp., Irvine, California, USA). The Qubit 2.0 Fluorometer (Life Technologies, Carlsbad, California, USA) was used for genomic DNA quantification. Finally, the genomic DNA was stored at −20°C until further analysis.

### 2.4 Molecular identification of *Klebsiella* using the *rpoB* gene

This study subjected the isolates to PCR with primer sets specific to *Klebsiella* targeting the *rpoB* gene. The PCR assay was conducted with the forward primer 5’-CAA CGG TGT GGT TAC TGA CG-3’ and the reverse primer 5’-TCT ACG AAG TGG CCG TTT TC-3’ (Bivand et al., 2024), yielding a 108 bp amplicon band size. The amplification reaction was carried out in a total volume of 25 μL that consisted of 10 µM of both forward and reverse primers, 12.5 μL of a 2X DreamTaq Green PCR Master Mix [4 mM MgCl_2_, loading buffer and 0.4 mM each of dATP, dCTP, mM dGTP, mMdTTP] (Thermo Fisher Scientific Inc., Waltham, MA, USA) and 5 ng of template DNA and nuclease-free water. The PCR conditions consisted of initial denaturation at 95°C for 5 minutes and then 35 cycles of denaturation at 94°C for 30 seconds, annealing at 55°C for 30 seconds, extension at 72°C for 30 seconds and a final extension at 72°C for 7 minutes. PCR products were identified by electrophoresis on 1% agarose gel stained with ethidium bromide and visualised under ultraviolet on a ChemiDoc imaging system (Bio-Rad ChemiDocTM MP Imaging System, Hertfordshire, United Kingdom). *K. pneumoniae* ATCC Type strain was used as a positive control, while RNase nuclease-free PCR water was used as a negative control.

### 2.5 Antimicrobial susceptibility testing for *Klebsiella* isolates

Antibiotic testing was carried out using the Kirby-Bauer disc diffusion method on Müller-Hinton (MH) agar plates. Pure cultures of isolates from faecal samples from sheep and water from the river stream were utilised to assess antibiotic resistance patterns. Nine different antibiotics from five distinct classes, including ampicillin (AMP; 10 μg), kanamycin (K; 30 μg), nalidixic acid (NA; 30 μg), cephazolin (KZ; 30 μg), ertapenem (ETP; 10 μg), meropenem (MEM; 10 μg), doripenem (DOR; 10 μg), and imipenem (IPM; 10 μg), were tested for antibiotic resistance on *Klebsiella* isolates following the Clinical and Laboratory Standards Institute (CLSI) antimicrobial susceptibility testing standards (CLSI, 2023). The MH plates were incubated at 37°C for 24 hours. After incubation, the isolates were classified as Resistant (R), Intermediate (I), or Susceptible (S) based on the size of the zone of bacterial growth inhibition as stipulated by the CLSI guidelines. Multidrug resistance (MDR) was defined as resistance to three or more classes of antibiotics tested (Magiorakos et al., 2012).

### 2.6 Library preparation and sequencing

Nine *Klebsiella* species isolates from sheep faeces and river stream were whole genome sequenced for downstream genomic analysis. Genomic DNA from *K. pneumoniae* isolates (n = 2) underwent library preparation with the ligation sequencing gDNA-Native barcoding kit (SQK-NBD114.96) from Oxford Nanopore Technology (ONT). Initially, the DNA was repaired using the FFPE DNA repair mix, followed by end preparation with the NEBNext Ultra II End Repair/dA-tailing Module from New England Biolabs (Teffera and Biolabs, 2019). The end-prepped DNA was then used for native barcode ligation employing the NEB Blunt/TA Ligase master mix and the NEBNext Quick Ligation Module in accordance with the manufacturer’s instructions. Subsequently, the barcoded DNA libraries were purified using Ampure XP beads, and native sequencing adapters were ligated. The libraries were further purified and quantified using the Qubit high-sensitivity kit from Thermo Fisher Scientific before being loaded for sequencing on the MinION 9.4.1 flow cell using the MinION 1KB platform from Oxford Nanopore. Library preparation for *K. pneumoniae* (n = 2), *K. variicola* (n = 2), and *K. michiganensis* (n = 5) was performed using the MGIEasy FS DNA Prep Kit (BGI, China) according to the manufacturer’s protocol. The prepared libraries were sequenced on the BGI MGISEQ-2000 platform (BGI Shenzhen, China) using a paired-end 150 nt strategy.

### 2.7 QC and bacterial genome assembly

Guppy base caller version 5.0.17 was employed to convert raw data in fast5 format into base-called data in fastq format. All reads with a quality score (Q) below 7.5 were excluded from subsequent data analysis. The quality of the trimmed data was evaluated using NanoPlot version 1.18.1 (De Coster et al., 2018). Quality filtering was performed using FiltLong version 0.2.0 (https://github.com/rrwick/Filtlong). The filtered Oxford Nanopore Technology (ONT) reads of *Klebsiella* strains were assembled *de novo* with Flye version 2.3.3 (Kolmogorov et al., 2019) and subsequently polished using the Medaka (https://github.com/nanoporetech/medaka) consensus pipeline. The quality of the sequenced reads generated by the MGI platform was assessed using FastQC software v0.10.1 (Andrews et al., 2010). Trimmomatic version 0.39 (Bolger et al., 2014) was used to trim ambiguous reads and remove sequenced adapters. The paired-end trimmed reads were assembled *de novo* using the MEGAHIT version 1.2.9 pipeline (Li et al., 2015). CheckM version 1.2.4 (Parks et al., 2015) was utilised to identify potential contaminants within assembled genomes. QUAST version 2.3 (Gurevich et al., 2013) was used to evaluate the draft genome assemblies of the strain. Subsequently, the assembled contigs were annotated employing the NCBI Prokaryotic Genome Automatic Annotation Pipeline (PGAAP) (Tatusova et al., 2016). Average Nucleotide Identity of the *Klebsiella* species was computed using skani v0.3.1 (Shaw and Yu, 2023) and visualized on R-studio v 4.5.1 using heatmap package. The taxonomic classification of bacterial strains was performed *in silico* using multilocus sequence typing (MLST) (Carattoli et al., 2014) and pathogenWatch (https://pathogen.watch/). Moreover, MLST from the Institut Pasteur (https://bigsdb.pasteur.fr/cgi-bin/bigsdb/bigsdb.pl?db=pubmlst_klebsiella_isolates&amp;page=plugin&amp;name=GenomeComparator) was used to type the sequenced genomes using the *Klebsiella oxycota* and *K. pneumoniae* complex databases.

### 2.8 Antibiotic resistance, virulence gene detection, and plasmid replicon determination

The ABRicate pipeline, together with AMRFinderplus v 4.0.23 (Feldgarden et al., 2021), was utilized to detect antibiotic resistance and virulent genes in the genomes of the nine *Klebsiella* spp. strains. Antibiotic resistance determinants were identified in the assembled genome using the ResFinder database, with minimum identity and coverage thresholds set at 70% each, respectively. The comprehensive antibiotic resistance database (CARD) was also consulted to identify antibiotic resistance genes. ABRicate v 1.0.0 was further utilised to identify efflux pump-coding genes and virulence factors in the sequenced genome, using the Virulence Factor Database (VFDB) with minimum identity and coverage thresholds of 70% each, respectively. Plasmid replicons were identified in the sequenced genomes using the plasmid finder database (Carattoli and Hasman, 2020) through ABRicate v.1.0.0. MobileOG-db v.2.0 (Brown et al., 2022) was also utilised to identify mobile genetic elements. The capsule types were identified using Kaptive (https://kaptive-web.erc.monash.edu/jobs), and sequence types were detected using Pathogenwatch (Pathogenwatch A Global Platform for Genomic Surveillance). Circos circular v0.63–10 data visualization software (Krzywinski et al., 2009) and chiplot (Ramírez et al., 2014) were used to visualise the profiles of antibiotic resistance genes (ARGS), virulence genes, and plasmid replicon types. Moreover, complete plasmids were generated using the Proksee server (https://proksee.ca). FastANI version 1.33 (Hernández-Salmerón and Moreno-Hagelsieb, 2022). and Clincker version 0.0.31 (Gilchrist and Chooi, 2021) were used to compare the plasmids of closely related replicons.

### 2.9 Pangenomics analysis

Pangenome analysis was conducted on the *K. pneumoniae* genomes determined using Roary v3.6.8 (Page et al., 2015) and Anvio-7.1 (Eren et al., 2015). Pangenome analysis was restricted to *K. pneumoniae* because it is the clinically dominant and most epidemiologically relevant species within the *K. pneumoniae* species complex (KpSC), particularly in relation to antibiotic resistance. A total of 84 downloaded *K. pneumoniae* genomes from 15 countries were retrieved and compared with the two sequenced *K. pneumoniae* strains T2 and T4 isolated from sheep faeces in South Africa (**Supplementary Table 1**). The selection was based on global genomes related to sequence types in this study as well asl including some of the South African genomes. The genomes were annotated using Prokka version 1.14 (Seemann, 2014) and Prodigal (Hyatt et al., 2010). For similarity searches between the coding domain sequences (CDSs) of assembled genomes Pairwise BLASTp and the Markov Cluster Algorithm (MCL) were utilized as described previously by Magome et al. (2024). The phylogenetic tree of the genomes was visualized using ITOL (Letunic and Bork, 2021) and FigTree v1.4.4 (Rambaut, 2014).

### 2.10 Whole genome data availability

The genome sequences of *K. pneumoniae, K. variicola* and *K. michiganensis* isolates used in this study have been deposited in the National Center for Biotechnology Information (NCBI) GenBank under accession numbers (JBIPSE000000000, JBIPSD000000000, JBBMKX000000000, JBBMLA000000000, JBBMKZ000000000, JBBMKY000000000, JBPPKQ000000000, JBPPKR000000000 and JBPPKS000000000).

## 3 Results

### 3.1 Identification and antibiotic resistance profiles of *Klebsiella* species

The SensiTitre system and PCR amplification of the *rpoB* gene identified these nine isolates as *Klebsiella* species. A total of two isolates from sheep faeces were confirmed as *K. pneumoniae*, two from the river stream as *K. variicola* and five from the river stream as *K. michiganensis*. These identifications were confirmed using PCR assays targeting the *rpoB* housekeeping gene, and whole genome sequencing.

*Klebsiella* isolates exhibiting resistance to three or more antibiotic classes were classified as multidrug resistant (MDR) (**Table 1**). All *Klebsiella* strains showed resistance to ampicillin, and high resistance rates were also observed to nalidixic acid (88.8%), kanamycin, and cephazolin (55.5%). Notably, no carbapenem resistance was detected in this study. All isolates exhibited MDR, harbouring resistance to three or more antibiotic classes (Motlhaping et al., 2025).

**Table 1:** Phenotypic antibiotic resistance patterns of *Klebsiella* spp. strains based on disc diffusion assay.

		*β*-lactam	Aminoglycoside	Quinolone	Cephalosporin	Carbapenem			
*Klebsiella* species	Isolates	AMP	K	NA	KZ	ETP	MEM	IPM	DOR
*K. variicola*	KMW3	**+**	**+**	**+**	**-**	**-**	**-**	**-**	**-**
	KMW4	**+**	**-**	**-**	**+**	**-**	**-**	**-**	**-**
*K. michiganensis*	KMW6	**+**	**+**	**+**	**-**	**-**	**-**	**-**	**-**
	KMW7	+	+	+	–	–	–	–	–
	KM20	+	+	+	+	–	–	–	–
	KM26	+	+	+	+	–	–	–	–
	KM23	+	–	+	+	–	–	–	–
*K. pneumoniae*	KPT2	**+**	**-**	**+**	**+**	**-**	**-**	**-**	**-**
	KPT4	**+**	**+**	**+**	**-**	**-**	**-**	**-**	**-**

AMP, Ampicillin; K, kanamycin; NA, nalidixic acid; KZ, cephazolin; ETP, ertapenem; MEM, meropenem; DOR, doripenem and IPM, imipenem.

### 3.2 Genomic features and assembly metrics of *Klebsiella* species

A total of 12 *Klebsiella* isolates were sequenced and assembled, comprising *Klebsiella michiganensis* (n = 5), *Klebsiella pneumoniae* (n = 3), and *Klebsiella variicola* (n = 4) (**Table 2**). Three genomes, KMTHO011 (*K. michiganensis*), KPHS11286 (*K. pneumoniae*), and KvF2R9T (*K. variicola*), served as references based on their completeness and availability in RefSeq. The total genome sizes ranged from 4.91 Mb to 6.55 Mb. Among *K. michiganensis* strains, KM26 (6.55 Mb) and KM20 (6.45 Mb) exhibited the largest genome sizes, while KM6, KM7, and KM23 had smaller genomes (5.22-5.33 Mb) but were the most fragmented, with 344 contigs and an N50 of 25.8 kb. In contrast, the reference strain KMTHO011 was highly contiguous, consisting of only two contigs with an N50 of 5.93 Mb and L50 of 1, consistent with high assembly quality. The *K. pneumoniae* strains, particularly KPHS11286, KPT2, and KPT4, showed high assembly continuity, with ≤7 contigs and N50 values exceeding 5.2 Mb. KPT4 and KPT2 had the highest predicted gene counts (7,851 and 7,359, respectively), in line with their relatively large genome sizes (~5.43 Mb). *K. variicola* isolates displayed moderate to high assembly variability. The reference genome KvF2R9T was assembled into a single contig, with an N50 of 5.52 Mb and L50 of 1, indicating a complete genome. In contrast, KvMW3 and KvMW4 exhibited draft assemblies (72 and 31 contigs, respectively) and smaller genome sizes (5.29 and 4.91 Mb). GC content across all isolates ranged from 55.1% to 58.0%, with species-specific trends observed. *K. michiganensis* strains exhibited lower GC content (55.1-56.5%), while *K. variicola* strains had the highest GC values (up to 58.0%). The number of protein-coding genes predicted by Prodigal ranged from 4,597 (KvMW4) to 7,851 (KPT4). Transfer RNA (tRNA) counts varied from 45 (KM23) to 88 (KPHS11286), with reference genomes generally showing higher tRNA content, reflecting their improved assembly completeness.

**Table 2:** Genome features of the assembled nine *Klebsiella* species sequenced in this study.

Contigs stats	KM20	KM23	KM26	KMTHO011*	KMW6	KMW7	KPHS11286*	KPT2	KPT4	KvF2R9T*	KvMW3	KvMW4
Total length	6,447,561	5,217,832	6,550,179	6,041,841	5,335,245	5,335,245	5,682,322	5,427,021	5,432,140	5,521,194	5,288,060	4,908,316
Num contigs	162	344	264	2	35	35	7	4	7	1	72	31
GC%	555	55.5	56.5	55.1	56.5	56.5	57.2	57.5	57.5	57.6	58	57.5
Num contigs > 100 kb	20	0	23	2	20	20	4	2	2	1	19	16
Num contigs > 50 kb	41	9	44	2	27	27	4	3	2	1	32	21
Num contigs > 20 kb	58	93	56	2	31	31	4	3	4	1	44	28
Num contigs > 10 kb	65	168	67	2	33	33	4	3	5	1	48	30
Num contigs > 5 kb	70	261	72	2	34	34	4	4	7	1	51	31
Num contigs > 2.5 kb	75	315	82	2	35	35	6	4	7	1	53	31
Longest contig	471.72	77.505	411.148	5,935,402	503.373	503.373	5,333,942	5,241,790	5,249,345	5,521,194	429.209	930.887
Shortest contig	207	1.5	200	106.439	4.773	4.773	1.308	8.92	6.515	5,521,194	1.501	5.812
Num genes (prodigal)	6.135	5.1	6.281	5.557	4.921	4.921	5.528	7.359	7.851	5.156	4.961	4.597
L50	12	68	14	1	8	8	1	1	1	1	11	5
L75	27	137	30	1	15	15	1	1	1	1	21	12
L90	43	217	45	1	23	23	1	1	1	1	33	18
N50	155,285	25,84	137,614	5,935,402	236,863	236,863	5,333,942	5,241,790	5,249,345	5,521,194	176,312	318,064
N75	81,609	12,841	79,853	5,935,402	124,429	124,429	5,333,942	5,241,790	5,249,345	5,521,194	94,536	160,257
N90	41,102	7,329	41,679	5,935,402	84,444	84,444	5,333,942	5,241,790	5,249,345	5,521,194	44,415	86,723
Transfer RNAs	84	45	83	83	64	64	88	86	87	85	73	70
Sequence Type	ST547	ST547^#^	ST547	ST817	ST860^#^	ST860^#^	ST11	ST5992^#^	ST5992^#^	ST2263	–	*-
Capsule Type	KL116	KL186	KL116	KL152	KL41	KL41	KL103	KL30	KL30	KL16	KL166	KL35

Kp, *K. pneumoniae*; Kv, *K. variicola*; Km, *K. michiganensis.* *Strains used as references based on completeness of the sequenced genomes available on refseq. ^#^refers to closest sequence type. (-) indicates untypeable sequence types.

### 3.3 MLST and capsule type of *Klebsiella* species

The *K. michiganensis* strains (KMW6 and KMW7) were identified as having the closest sequence type 860 (ST860), which best matches capsule KL41. *K. michiganensis* strain KM20 and KM26 are assigned sequence types that best match sequence type 547 (ST547), which best matches KL116, while strain KM23 has a closest ST of 547, with an unknown capsule type that best matches KL186. In addition, the *K. michiganensis* strain KMTHO011 used as a reference has an ST of 817 and which best matches KL152. Moreover, the *K. variicola* strain KvMW3 best matches capsule KL166, while strain KvMW4 best matches KL41. The reference *K. variicola* strain KvF2R9T was assigned sequence type 2263 (ST2263) and capsule type KL16. The two sequenced *K. pneumoniae* strains, KPT2 and KPT4, present with STs, that are closely related to ST5992, and both have the capsule type KL30. Their closest STs are *K. pneumoniae* strains DAFUVP011 and DAJMOO011, both classified as ST5992 and isolated from humans in Switzerland and Finland, respectively. The reference strain KPHS11286 was assigned sequence type 11 (ST11), which best matches capsule KL103 (**Table 2**).

### 3.4 Average nucleotide identity of *Klebsiella* species

**Figure 1:**
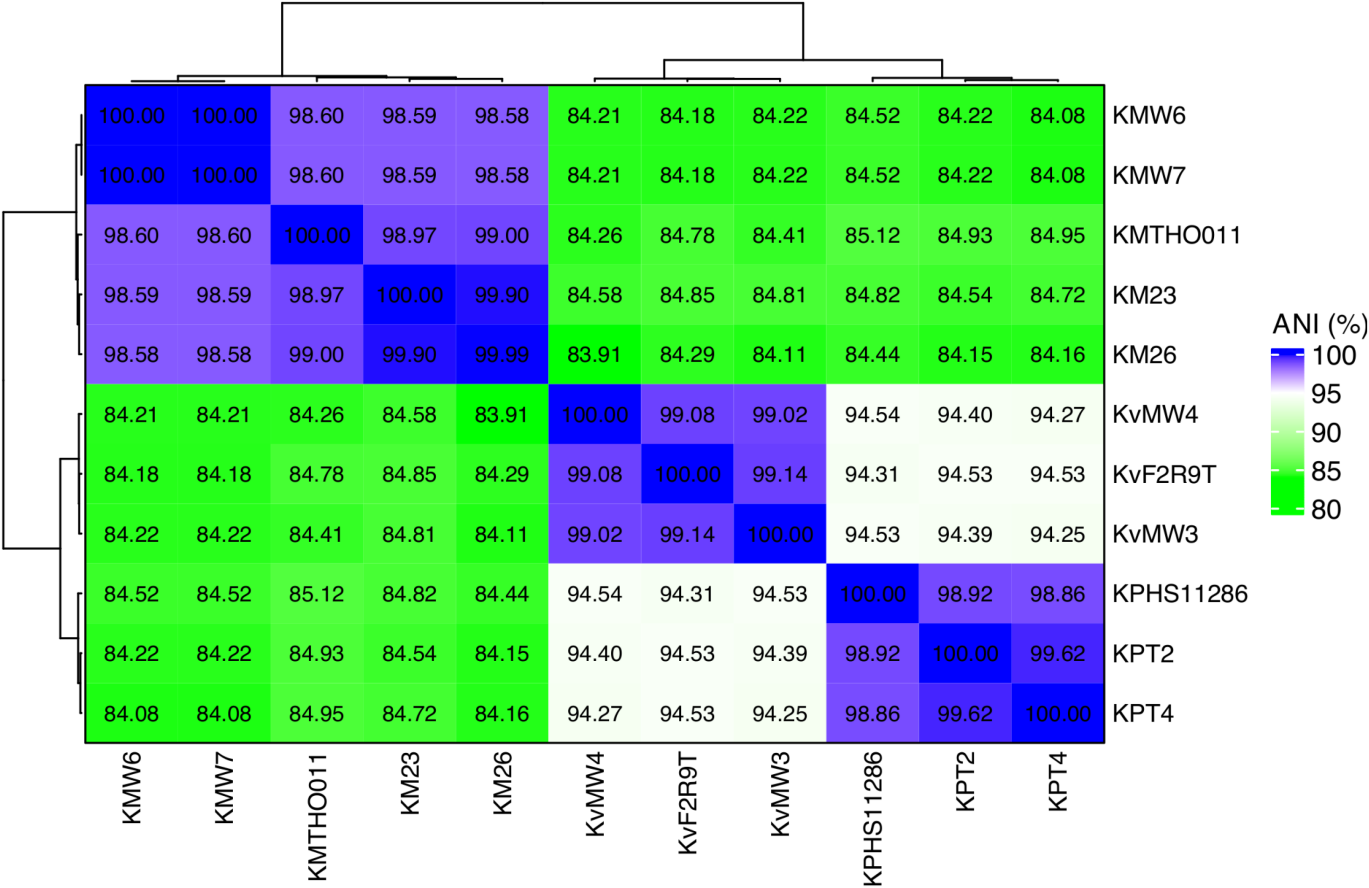
Average nucleotide identity (ANI) of the sequenced *Klebsiella* species strains. Strains KMTHO011, KvF2R9T and KPHS11286 were used as references based on the completeness of the sequenced genomes available on RefSeq. KP represents *K. pneumoniae*, while Kv as *K. variicola*, and Km as *K. michiganensis*.

The average nucleotide identity (ANI) analysis in 12 *Klebsiella* genomes revealed clear genomic distinctions among the *Klebsiella* isolates (**Figure 1**). The *K. michiganensis* strains KM20 and KM26 showed extremely high ANI values (> 99.8%), indicating they are nearly identical and likely belong to the same strain or a closely related lineage. The *K. michiganensis* strain KM23 also clustered closely with strains KM20 and KM26, sharing over 99% ANI, forming a tight group that likely represents a clonal lineage. In contrast, *K. michiganensis* strain KMTHO011, KMW6, and KMW7 formed a separate cluster, showing ANI values ranging between 86-87% compared to the *K. michiganensis* group. Another distinct group consisted of *K. pneumoniae* strains KPHS11286, KPT2, and KPT4, which exhibited high within-group ANI values of 85%-100% but much lower similarity to the *K. michiganensis* groups, confirming their genomic distinctness. Similarly, *K. variicola* strain KvF2R9T grouped with strains KvMW3 and KvMW4, sharing moderate to high ANI values (80-100%) within the cluster, yet remaining distinct from the clinical isolates. These findings, visually supported by the heatmap in **Figure 1**, demonstrate at least four major genomic groups among the *Klebsiella* isolates, highlighting both species-level diversity and potential clonal relationships within *Klebsiella* populations.

### 3.5 Identification of virulence genes in the *Klebsiella* species

**Figure 2:**
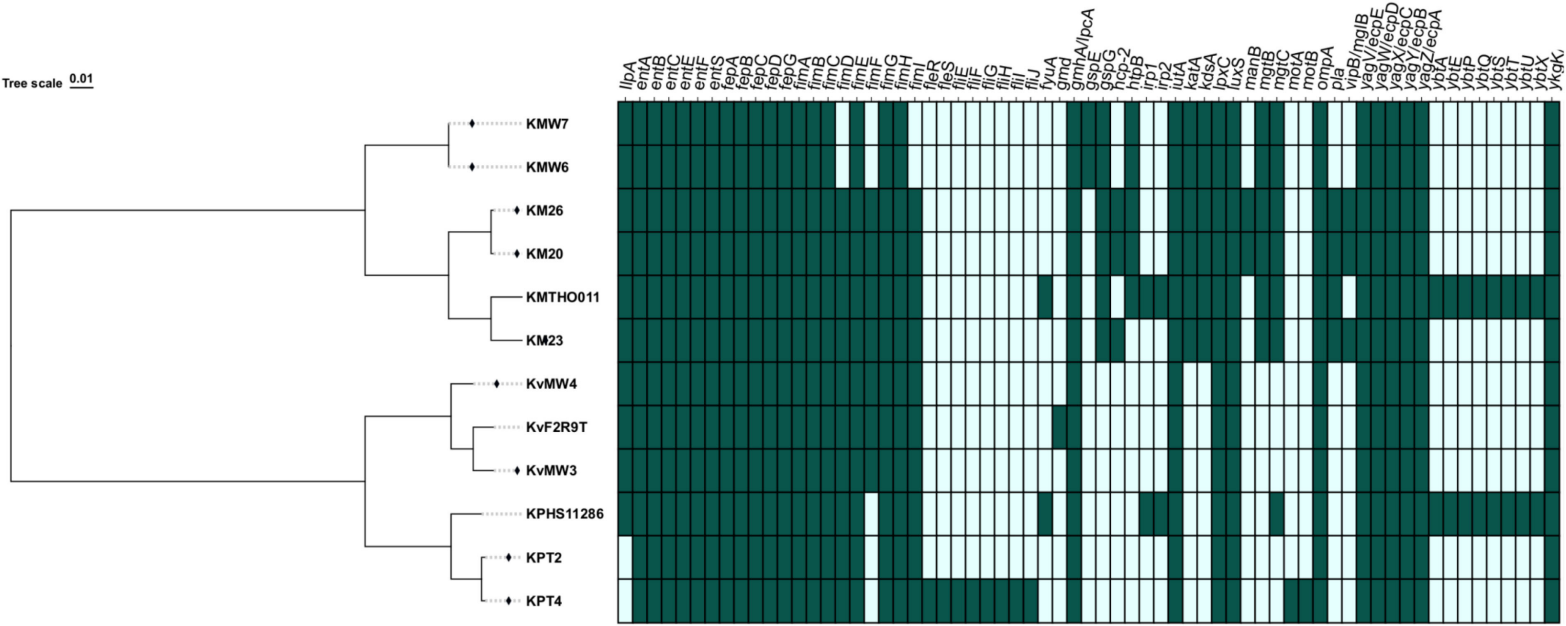
Virulence genes identified among *Klebsiella* species: Dark green colour= presence of the genes; Light green colour=absence of the gene.

The nine *Klebsiella* spp. strains were analysed for virulence genes (**Figure 2**). The *K. pneumoniae* strains KPT2 and KPT4 from sheep contained 30 virulence genes linked to adhesin and invasion (*ompA*), as well as enterobactin for iron absorption (*entA, entB*, *entC, entD, entE, entF, entS*, *fepA*, *fepB*, *fepC* and *fepG*), fimbrial adhesin (*fimA, fimB, fimC, fimD, fimE, fimF, fimG, fimH, fimI, yagV/ecpE, yagW/ecpD, yagX/ecpC, yagY/ecpB, yagZ/ecpA* and *ykgK/ecpR*), LPS core biosynthesis (*gmhA/lpcA)*, Iron uptake (*iutA*), Lipid A synthesis (*lpxC*) and Quorum sensing via AI-2 (*luxS*). Strain KPT4 exhibited 10 additional flagellar virulence genes: *fleS, fleR, fliE, fliF, fliG, fliH, fliI, fliJ, motA*, and *motB*. The shared virulence genes between strains KPT2 and KPT4 encoded proteins for adhesin, invasion, enterobactin, iron absorption, and adhesion.

Each of the *K. variicola* strains, KvMW3 and KvMW4, isolated from a river stream, harboured 32 virulence-associated genes. These included genes involved in iron acquisition (e.g., *entA*, *entB*, *entC*, *entE*, *entF*, *entS*, *fepA*, *fepB*, *fepC*, *fepD*, *fepG*), fimbrial assembly (*fimA*–*fimI*), outer membrane integrity (*ompA*), quorum sensing (*luxS*), and other functions such as lipopolysaccharide biosynthesis (*gmhA/lpcA*, *lpxC*) and siderophore uptake (*iutA*). Additional genes associated with the *ecp* operon (*yagV/ecpE* to *ykgK/ecpR*) were also consistently present.

In contrast, *K. michiganensis* strains KMW6 and KMW7 carried 36 virulence genes, encompassing all those found in KMW3 and KMW4, with the addition of genes such as *gspE*, *gspG*, *htpB*, *katA*, *kdsA*, *mgtB*, and *mgtC*, suggesting enhanced stress response and secretion capabilities. Strains KM20 and KM26 of *K. michiganensis* exhibited the most extensive virulence gene repertoire, with 42 genes, including those found in KMW6 and KMW7, as well as *hcp-2*, *manB*, *pla*, and *vipB/mglB*, indicating potential for increased pathogenicity and environmental adaptability. KM23 harboured 40 virulence genes, lacking only *htpB* and *manB*, compared to KM20 and KM26. Across all genomes analysed, 29 virulence genes were universally present (100%), including *entA*–*entF*, *fepA*–*fepG*, *fimA*–*fimH*, *gmhA/lpcA*, *iutA*, *lpxC*, *luxS*, *ompA*, and the *ecp* cluster (*yagV* to *ykgK*). These genes can be grouped into functional clusters such as iron acquisition, fimbrial assembly, membrane integrity, and secretion systems. Conversely, the least prevalent genes, *fleR*, *fleS*, *fliE*, *fliF*, *fliG*, *fliH*, *fliI*, *fliJ*, *gmd*, *motA*, and *motB* were detected only in strain *K. pneumoniae* KPT2, suggesting limited distribution or strain-specific roles.

### 3.6 Identification of antibiotic resistance genes in the *Klebsiella* species

**Figure 3:**
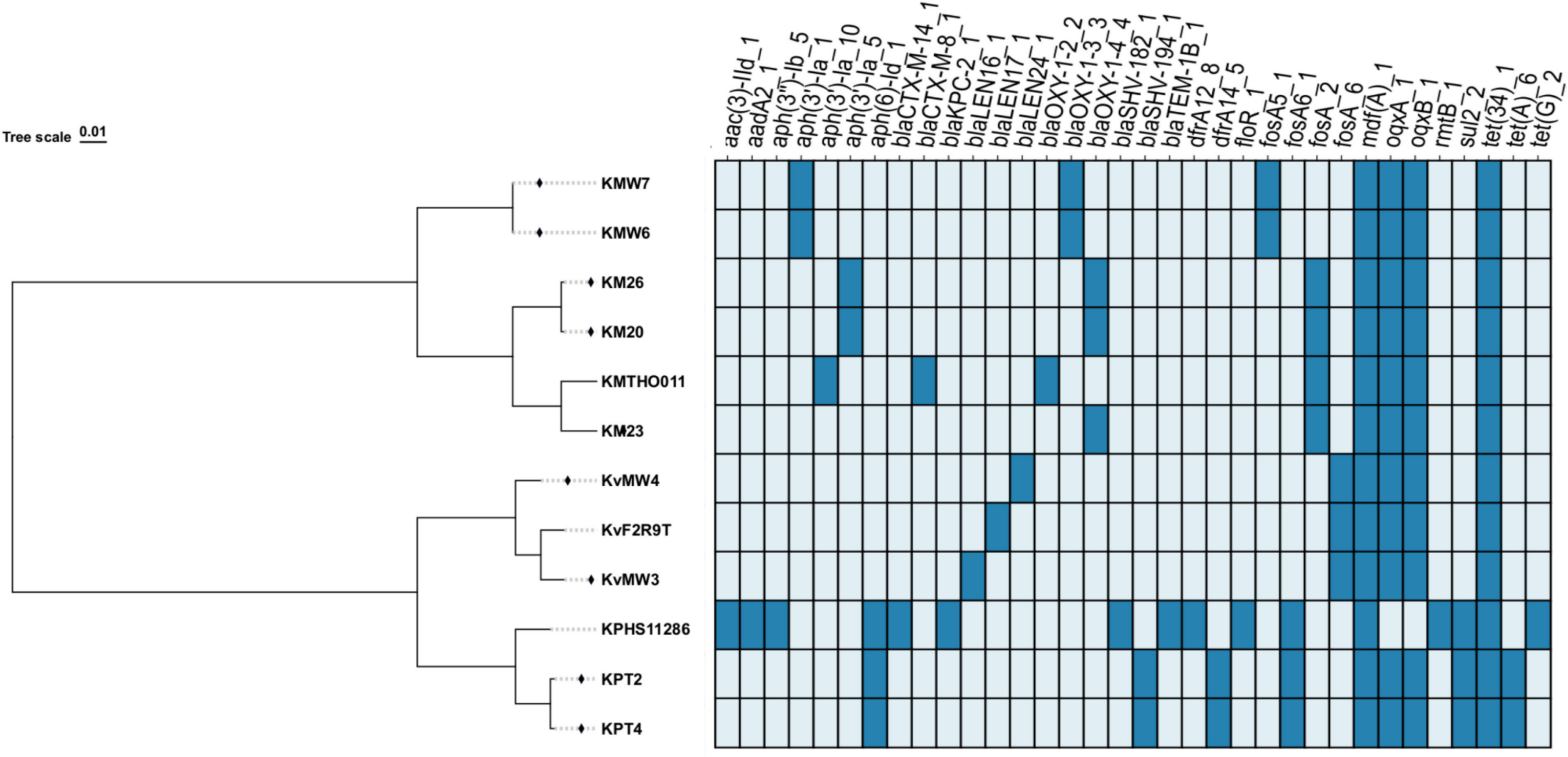
Antibiotic resistance genes identified among *Klebsiella* species: Dark blue colour= presence of the genes; Light blue colour=absence of the gene; ARGs=Antibiotic resistance genes.

Analysis of antibiotic resistance genes (ARGs) in the nine *Klebsiella* genomes (**Figure 3**) reveals that *K. pneumoniae* strains KPT2 and KPT4 harbour 10 ARGs. The core ARGs identified in both strains include *tet(A)_6* and *tet(34)_1*, which encodes tetracycline resistance; *sul2* (sulphonamide resistance); *dfrA14_5* (trimethoprim resistance)*; aph(6)-id* (aminoglycosides resistance)*; bla*_SHV-194_ (β-lactamase production)*; fosA6* (fosfomycin resistance)*; oqxB* and *oqxA* (quinolones resistance); and *mdf(A)_1* (multidrug efflux pump).

In contrast, the *K. variicola* strains from the river stream contained 6 ARGs, including *oqxB, oqxA, fosA, mdf(A), tet34* and either *bla*_LEN24_ or *bla*_LEN16_. In addition, the *K. michiganensis* strains KM20 and KM26 exhibited 7 ARGs, which included *aph(3’)-Ia_5, blaOXY-1-4_4, fosA, mdf(A)_1*, *oqxB, oqxA* and *tet(34).* The *K. michiganensis* strain KM23 exhibited all genes present in strain KM20 and KM26 except for the *aph(3’)-Ia* gene. Furthermore, the *K. michiganensis* strains KMW6 and KMW7 harboured 7 ARGs. The core ARGs identified in both *K. michiganensis* strain KMW6 and KMW7 include *aph(3’)-Ia_1, bla*_OXY-1-3_*, fosA5, mdf(A), oqxA, oqxB* and *tet(34).* Among the examined sequenced genomes, the most prevalent ARGs identified were *mdf(A)_1* and *tet(34)_1*, present in 100% (n = 12) of the strains, including the reference strains used in this study. The second most common ARGs were detected in 92% (n = 11) of strains, including *oqxA* and *oqxB*. The least ARGs identified included *aac(3)-IId_1, aadA2_1, aph(3’’)-Ib_5, aph(3’)-Ia_10, bla*_CTX-M-14_1_*, bla*_CTX-M-8_1_*, bla*_KPC-2_1_*, bla*_LEN16_1_*, bla*_LEN17_1_*, bla*_LEN24_1_*, bla*_OXY-1-2_2_*, bla*_SHV-182_1_*, bla*_TEM-1B_1_*, dfrA12_8, floR_1, rmtB_1* and *tet(G)_2*, which were present in 8% (n = 1) of the genomes examined.

### 3.7 Plasmid replicon types across 12 *Klebsiella* species strains

**Figure 4:**
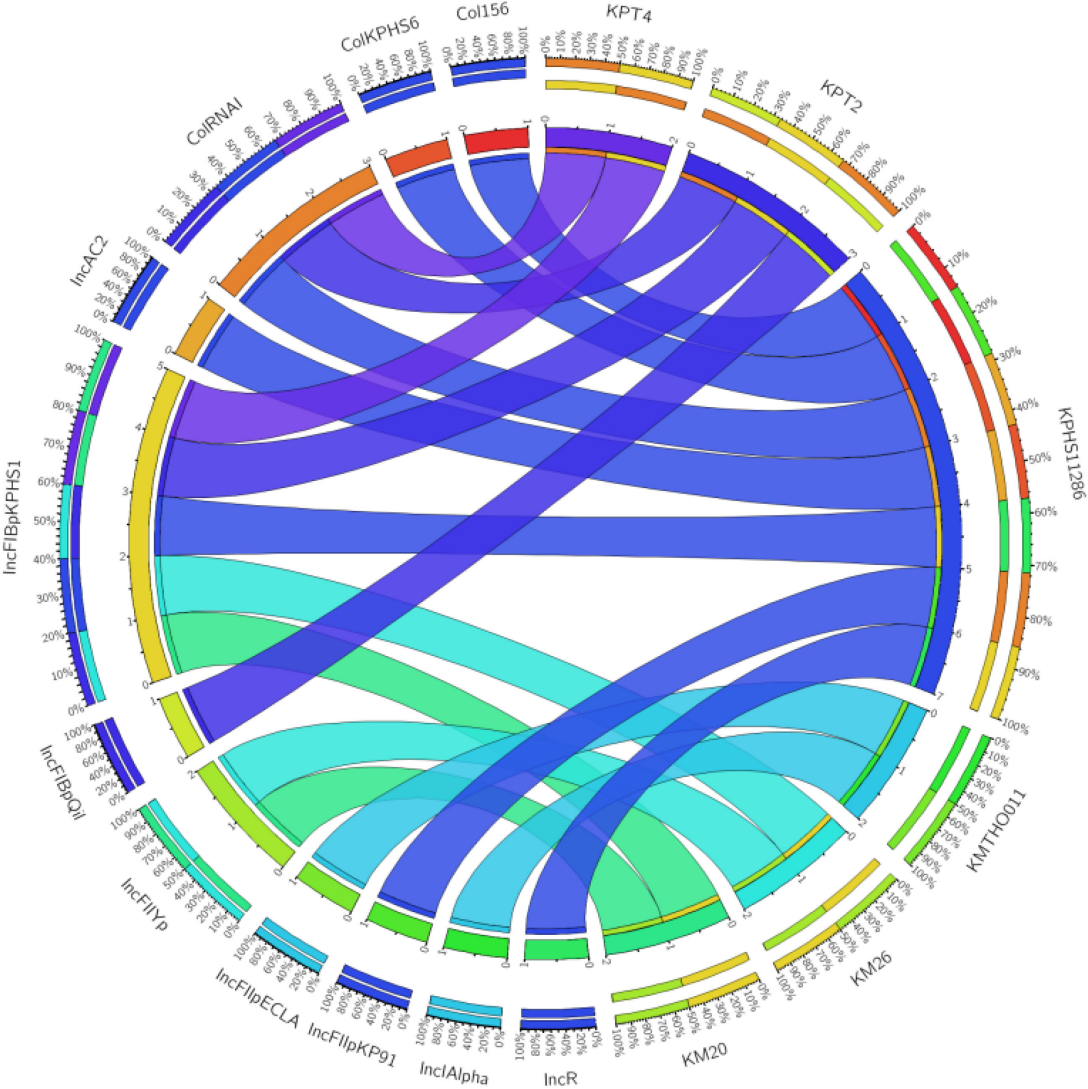
Circos plots of the plasmid replicon types in *Klebsiella* species genomes. Kp, *K. pneumoniae* and Km, *K. michiganensis*.

The plasmid replicon types were analysed across 12 genomes of *Klebsiella* species, including the nine sequenced strains compared with the reference strains used for ANI (**Figure 1**). A total of 11 plasmid replicon types were identified across all the *Klebsiella* spp. genomes (**Figure 4**). In both *K. pneumoniae* strains KPT2 and KPT4, two plasmid types were detected: *ColRNAI_1* and *IncFIB(pKPHS1_1_pKPHS1)*, with strain KPT2 also harbouring the additional replicon type *IncFIB(pQil_1_pQil).* In *K. michiganensis* strain KM20 and KM26, two plasmid replicon types were detected, which included *IncFIB(pKPHS1_1_pKPHS1* and *IncFII(Yp)_1.* No replicon types were detected in *K. variicola* strains KvMW3 and MW4, and in *K. michiganensis* strains KMW6, KMW7, and KM23. Among the genomes examined, the most prevalent plasmid type identified was *IncFIB (pKPHS1_1_pKPHS1)*, present in 41% (n = 5) of the strains, including *K. michiganensis* strains KM20 and KM26, as well as *K. pneumoniae* strains KPT2, KPT4 and KPHS11286. The second most common plasmid type *ColRNAI_1* was detected in 25% (n = 3) strains, exclusive to *K. pneumonaie* strains KPT2, KPT4 and KPHS11286.

### 3.8 Plasmid genetic features of *K. pneumoniae* strains KPT2 and KPT4

Three plasmids (AC125, AA023, and AB595) were identified in *K. pneumoniae* strain KPT2, and two plasmids (AC125 and AB595) in *K. pneumoniae* strain KPT4 (**Table 3**). No plasmids were detected in other *Klebsiella* species. The 110 kbp plasmid AC125 found in both strains comprises *ColRNAI* and *IncFIB(pQil)* replicon types, with a GC content of 48.97%. This plasmid contains 152 coding sequences (CDS) and two transfer RNAs (tRNA 75 bp) along with a significant number of mobile genetic elements (MGEs). The annotated MGEs include 42 genes, which consist of (n = 1) integration/excision, (n = 10) replicon/recombination/repair, (n = 22) phages, (n = 7) stability/transfer/defence, and (n = 2) transfer in strain KPT2 (**Supplementary Figure 1A**). The plasmid lacks antibiotic resistance genes. Similarly, many MGEs were identified in plasmid AC125 of *K. pneumoniae* strain KPT4, which also lacks ARGs (**Supplementary Figure 1B**). The plasmid exhibits 98.3% identity with *K. pneumoniae* strain CRKP-35’s unnamed plasmid (accession CP107355.1), according to ANI. The carbapenem resistance gene (*bla*_KPC-2_) is present in strain CRkp-35’s unnamed plasmid, whereas this gene is absent from the plasmids of strains KPT2 and KPT4 (**Supplementary Figure 1C**).

**Table 3:** Plasmid features identified in *Klebsiella pneumoniae* strains KPT2 and KPT4 isolated from sheep faeces.

	*K. pneumoniae* strain KPT2			*K. pneumoniae* strain KPT4	
Plasmids	AC125	AB595	AA023	AC125	AB595
Size	110000 bp	8000 bp	65000 bp	110000 bp	8000 bp
GC content	48.97%	54.98%	52.53%	48.97%	54.97%
Replicon types	*IncFIB(pQil)_1_pQil*	*IncFIB*	*IncFIB*	*IncFIB*	*IncFIB*
	*ColRNAI*				
Coding sequences (CDS)	152	12	86	160	12
tRNA	2(75 bp)	0	0	2 (75 bp)	0
Antibiotic resistance genes (ARGs) present	No ARGs	*aph(6)-Id*	No ARGs	No ARGs	*aph(6)-Id*
		*dfrA14*			*tetR*
		*sul*			*tet(A)*
		*tetR*			*sul*
		*tet(A)*			
	
Total mobile genetic elements (MGEs)	42	3	31	53	2

The plasmid AB595, identified in these two strains, is 98% similar and consists of the replicon-type *IncFIB* with a GC content of 54.98% (**Table 3**). This plasmid contains 12 coding sequences (CDS). The plasmid AB595 in strain KPT2 has three mobile genetic elements annotated as integration/excision genes (*tnpA*) and *exc1* transfer genes (**Supplementary Figure 2A**). It carries aminoglycoside *aph(6)-Id*, trimethoprim *dfrA14*, tetracycline *tetR* and *tetA*, and sulphonamide *sul*2 resistance genes (**Supplementary Figure 2A**). Plasmid AB595 in *K. pneumoniae* strain KPT4 contains 12 coding sequences (CDS), with two mobile genetic elements identified (**Supplementary Figure 2B**). This plasmid harbours ARGs, including *aph(6)-Id, sul2, and* three copies of *tetA*. The gene orientation in this plasmid places *dfrA14* and *aph(6)-Id* in close proximity. Based on ANI, the closest related plasmid AB595 is *Shigella sonnei* strain 202108535-plasmid_p202108535-7 (accession OP038299:1-8375), which consists of ARGs such as *sul2, dfrA14*, and *aph(6)-Id*. However, the *dfrA14* gene’s percentage identity and positioning differ within the plasmid AB595 cluster of the two sequenced genomes in this study (**Supplementary Figure 2C**). Other hypothetical protein genes are present before the *aph(6)-Id* gene in strain KPT2. The *aph(6)-Id* is more conserved and identical (100%) among these compared plasmids.

A 65-kbp plasmid AA023 was identified exclusively in the *K. pneumoniae* strain KPT2, which boasts a GC content of 52.53%. This plasmid carries an *IncFIB* replicon with no ARGs detected. It comprises 86 coding sequences (CDS) and 31 MGEs, including (n = 11) for integration/excision, (n = 5) for replication/recombination/repair, (n = 14) for stability/transfer/defence, and (n = 1) for transfer (**Supplementary Figure 3**). This plasmid shows an ANI greater than 95.5%, identical to plasmid AA022 (CP133155.1) from *K. variicola* strain T2. Plasmid AA022 was isolated from wastewater treatment plant B in the North West Province of South Africa.

### 3.9 Pangenomes and average nucleotides identity of *K. pneumoniae* genomes

**Figure 5:**
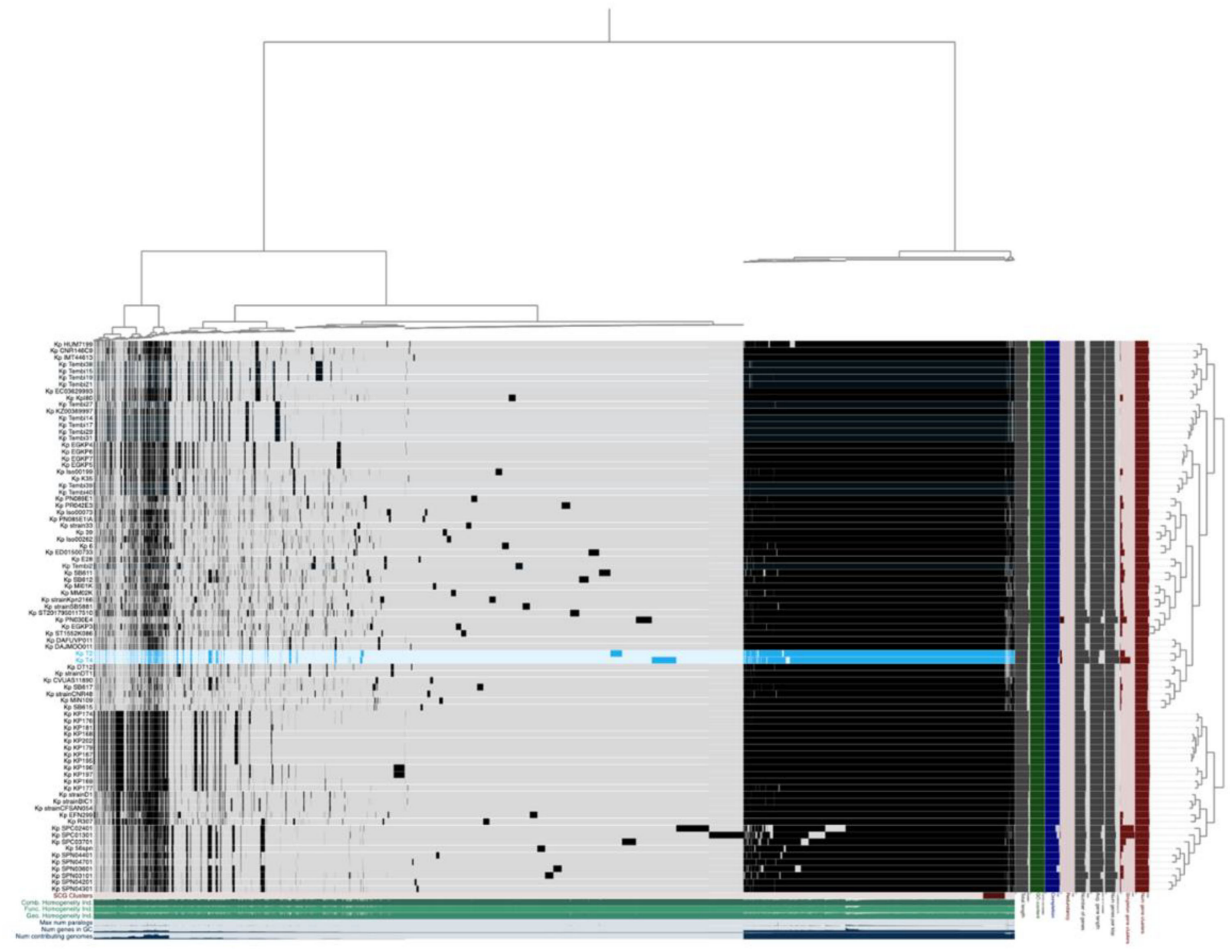
Global pangenome of the *K. pneumoniae* strains (n = 86), including the two sequenced strains highlighted in blue in this study visualized using ANVI’O. Based on core genes, the layers depict individual genomes arranged according to their phylogenomic relationships. The pangenome was computed using 34305 gene clusters found across the genomes. Dendrogram clustering of samples is ordered by gene cluster presence/absence. Items order: presence absence (D, Euclidean; L, Ward).

The pangenome of the 86 K*. pneumoniae* genomes, including the two sequenced strains, (highlighted in blue in **Figure 5**), consist of 34–305 total gene clusters. The analysis of dispensable genomes was conducted using the Roary and Anvi’o tools. This collection includes genomes from 23 South African strains, 22 from other African countries, and the rest from non-African countries (**Supplementary Table 1**). Roary identified pangenome clusters shared across all strains, classifying 229 gene clusters as core genes present in all strains. About 2553 gene clusters were categorized as soft-core genes, found in 95-99% of strains, while 3819 clusters were classified as shell genes, which are present in some strains. The majority of the gene clusters, totalling 27,704, were identified as cloud genes, which are found in only a few strains. Across the 86 genomes, 34305 genes were observed using the Anvi’o tool. Several accessory genes were identified in the sequenced T4 strain, including *fliE, fliF, fliG, fliH, fliI, fliJ, fleS* and *fleR*, along urease alpha subunit and including hypothetical proteins. Average nucleotide identity (ANI) analysis showed that the South African strains T2 and T4 had 98% genetic similarity, as they were isolated from two different sheep from the same farm. These strains clustered with *K. pneumoniae* strains DAFUVP011 and DAJMOO011, isolated from humans in Switzerland and Finland, respectively, which also share an ANI of 98%.

**Figure 6:**
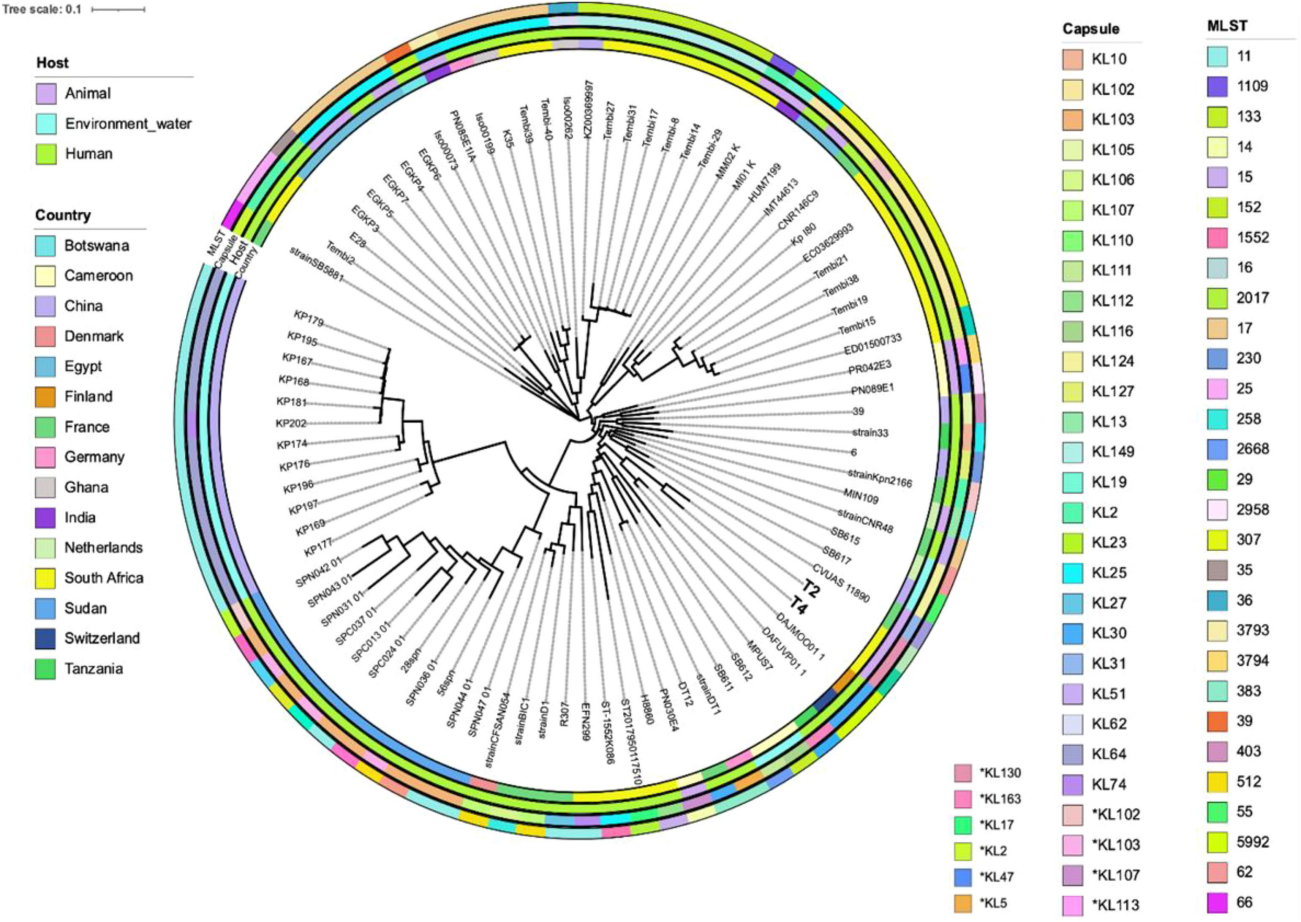
Classification of MLST sequence and capsule types identified in the 86 compared *K. pneumoniae* strains including the two sequenced strains in this study. The phylogenetic tree is based on maximum likelihood computed from pangenome analysis. The inner circular ring represents the country, followed by host, capsule types and sequence types. * Indicated closest capsule types.

Phylogenetic analysis based on pangenomics analysis revealed that the South African strains (Tembi40 and Tembi39) shared the KL25 capsule type, while strains from Egypt (EGKP5, EGKP6, EGKP7, and EGKP4), Ghana (Iso00199) and China (K35), grouped together, designated as ST-17 with KL25 capsule type (**Figure 6**). Multilocus sequence typing (MLST) analysis of the 86 K*. pneumoniae* genomes revealed 45 distinct sequence types (STs). The most prevalent sequence type was ST11 (n = 16), commonly found in Sudan, France, China and Ghana. This was followed by ST307 (n = 8) from South Africa, France and Germany, and ST152 (n = 7) from South Africa and China. A total of 35 capsule types were identified, while 10 capsule types were classified as unknown (**Figure 6**). The most common capsule type, KL64 (n = 11), was predominantly found in water-derived isolates from China and was associated with ST11. Other prevalent capsule types included KL25 (n = 9), followed by KL102 (n = 8), KL149 (n = 7), and KL103 (n = 7), observed in countries such as Germany, France, Botswana, Sudan, South Africa, Egypt, Ghana, and China. Several South African genomes, isolated from human and animal strains (Tembi15, Tembi38, Tembi19 and Tembi21), were assigned the KL102 capsule type, which is associated with ST307. Other South African strains (Tembi27, Tembi31, Tembi7, Tembi8, Tembi14, and Tembi29) exhibited the KL149 capsule type, belonging to ST152. ST307 was also found in other countries, such as Germany (strain IMT44613) and France (strain CNR146C9). The two sequenced South African strains, T2 and T4, presented capsule type that best matched KL130 (**Figure 6**).

## 4 Discussion

This study identified and characterized nine *Klebsiella* isolates recovered from sheep faeces and river stream samples in a communal village in the North West Province of South Africa. Through integrated genomic and phenotypic analyses, the study highlights the role of rural agricultural environments as reservoirs and potential amplifiers of antimicrobial resistance (AMR) and virulence determinants. The isolates were taxonomically classified as *Klebsiella pneumoniae*, *K. variicola*, and *K. michiganensis*, reflecting both species-level diversity and evidence of clonal expansion within the local ecosystem. The detection of *K. pneumoniae* in sheep faeces and *K. variicola* and *K. michiganensis* in adjacent river streams underscores the close interface between livestock and environmental compartments in rural settings. Such environments, where untreated water is commonly used for livestock, human consumption, and irrigation, facilitate microbial exchange and may promote the dissemination of pathogenic and resistant strains. Notably, the identification of clonally related *K. michiganensis* strains (KM20, KM23, and KM26), sharing >99% average nucleotide identity (ANI), across distinct water samples suggests environmental persistence and potential for long-term survival and transmission. These findings emphasize *Klebsiella* species’ ecological adaptability and zoonotic potential in rural agricultural landscapes. This study significantly contributes to understanding *Klebsiella* species in small ruminant-associated environments in South Africa by applying whole-genome sequencing (WGS). The study offers a comprehensive genomic and phenotypic characterization encompassing the resistome, virulome, plasmid content, and phylogenetic relationships by examining isolates from sheep faeces and adjacent river streams.

The use average nucleotide identity (ANI) analysis of *Klebsiella* genomes revealed clear genomic groupings reflecting species-level differences and possible clonal relationships. *K. michiganensis* strains KM20 and KM26 were nearly identical, sharing over 99.8% ANI, strongly suggesting they are part of the same clonal lineage. The *K. michiganensis* strain KM23 also grouped closely with these two strains, showing more than 99% similarity, which points to a shared evolutionary background (Hernández-Salmerón and Moreno-Hagelsieb, 2022). On the other hand, *K. michiganensis* strains KMTHO011, KMW6, and KMW7 showed much lower similarity (86-87% ANI) when compared to strain KM20 and KM26, indicating notable genomic divergence. This is consistent with earlier studies reporting high genomic plasticity and the presence of multiple *K. michiganensis* lineages in both environmental and clinical contexts (Simoni et al., 2023; Yamada et al., 2024). The *K. pneumoniae* strains KPHS11286, KPT2, and KPT4 formed a distinct genomic cluster, sharing high similarity (85-100%ANI), yet clearly separating from the *K. michiganensis* group, confirming species-level differentiation (Liu et al., 2012; Lekota et al., 2024). They highlight the value of ANI-based genomic surveillance for distinguishing between *Klebsiella* species and tracking clonal lineages, particularly in complex environments where different strains may circulate silently.

Strains T2 and T4 were identified as non-hypervirulent *K. pneumoniae* strains, though they carried virulence genes related to invasion, enterobactin, capsule formation, iron-absorption and adhesive, which are key factors for pathogenesis in “One Health” settings (Monteiro et al., 2024). The two strains, T2 and T4, had >98% ANI, indicating they were isolated from two different sheep on the same farm. Based on phylogenomic placement, they group closely with isolates DAFUVP011 and DAJMOO011, isolated from humans in Switzerland and Finland, respectively. All four strains mentioned above were defined with ST5992. This study reports the first sequence type identified in livestock, and it has not been reported in South Africa in other hosts. It is therefore evident that this sequence can also be found in humans, as demonstrated by comparative genomics in this study.

Pangenomic analysis of *K. pneumoniae* strain KPT4 revealed the presence of unique accessory genes, including hypothetical proteins and the urease alpha subunit, which are essential for survival in diverse environments (Duran Ramirez et al., 2022). Additionally, genes associated with flagellar motor assembly (*fliE, fliF, fliG, fliH, fliI*, and *fliJ*) and motility (*motA* and *motB*) were identified, demonstrating their roles in biofilm formation and survival under antibiotic stress (Sharma et al., 2019). Such environmental fitness genes improve the survival and persistence of these bacteria in shared water sources and animal habitats, heightening the potential for cross-species transmission and chronic contamination in “One Health” contexts. This study showed that *bla*_SHV-194,_ which confers *β*-lactamase resistance, was present in the two *K. pneumoniae* genomes studied and is commonly associated with ampicillin resistance in *K. pneumoniae*. This gene is underreported among South African *K. pneumoniae* strains, particularly among ESBL-producing strains. However, the gene was reported in 91.4% of *K. pneumoniae* strains from several hospitals in northern Iran (Farhadi et al., 2021).

Whole-genome sequencing of *K. pneumoniae* isolates revealed the presence of multiple antibiotic resistance genes (ARGs) located on plasmids, notably plasmid AB595. The co-localization of these genes on plasmid AB595 suggests its potential role as a multidrug resistance vector, particularly if it possesses conjugative capabilities (Khedkar et al., 2022). Mobile genetic elements (MGEs), often adjacent to ARGs, facilitate horizontal gene transfer and stable maintenance within bacterial populations (San Millan, 2018; Stephens et al., 2020). In this study, plasmid AB595 harboured such MGEs, supporting its role in resistance dissemination. Notably, *aph(6)-Id* and *sul2* were found within the same plasmid or operon, indicating a genetic linkage that may enhance co-transfer and expression (Venkatesan et al., 2023). Comparative analysis revealed that AB595 shares structural similarity with plasmid p202108535–7 from *Shigella* 202108535 (Lefèvre et al., 2023) and plasmid pABC-3 (Miranda et al., 2016), suggesting interspecies plasmid exchange. The presence of such multidrug-resistant plasmids in *K. pneumoniae* from livestock and environmental water sources poses a significant public health risk, particularly in rural settings where direct contact, contaminated water, or food may facilitate transmission, an issue of concern within the “One Health” framework. The *tet(A)* gene, carried by AB595, encodes an efflux pump that actively expels tetracycline from bacterial cells, reducing intracellular antibiotic concentrations and promoting survival (Xu et al., 2021; Wang et al., 2024). This mechanism is especially concerning in rural areas where tetracyclines are widely used in human and veterinary medicine, increasing the likelihood of resistance selection. Furthermore, the *dfrA14* gene, conferring resistance to trimethoprim, was identified in strains KPT2 and KPT4. Although detected in only 17% of *Klebsiella* strains in this study, *dfrA14* is more prevalent in clinical isolates, often co-occurring with *sul2* (Xu et al., 2021). Its global emergence following the introduction of trimethoprim into bacterial populations underscores the need for continued surveillance and molecular characterization of resistance determinants in environmental and animal reservoirs.

The primary plasmid replicon types belong to the diverse incompatibility (*Inc*) family, primarily affecting *Enterobacteriales* (Chen et al., 2024). Plasmid AC125 showed 98.3% identity to an unnamed plasmid in *K. pneumoniae* strain CRkP-35 (Hu et al., 2023), suggesting that AC125 may be a prophage plasmid rich in MGEs, particularly in prophage regions. Similar plasmids lacking ARGs have been reported, such as the P3 prophage plasmid found in *K. pneumoniae* strains from patients diagnosed with relapsed acute myeloid leukaemia (Yoshino et al., 2021). A relatively exclusive plasmid AA023 was identified in strain KPT2, exhibiting 95.55% identity to plasmid AA022 (CP133155.1) found in *K. variicola* strain KPT2, isolated from the North-West Province, South Africa. Both AA022 and AA023 appear to be related phage-like plasmids with minimal ARGs and encode many uncharacterised proteins. Both plasmids contain over 90% hypothetical proteins with unknown functions.

The antimicrobial resistance (AMR) gene profiles identified in *Klebsiella* species from water sources associated with small livestock farming highlight the environmental persistence and potential spread of multidrug-resistant *Klebsiella* strains. The presence of *oqxAB* efflux pumps is particularly concerning, as these genes are associated with reduced susceptibility to last-resort antibiotics such as tigecycline and fluoroquinolones, and have been frequently detected in environmental reservoirs, including swine manure, hospital wastewater, and aquatic systems (Wyres and Holt, 2018; Wang et al., 2019; Yang et al., 2024). In contrast, while *bla*_LEN_ variants naturally occur in *K. pneumoniae*, their association with resistance to last-resort antibiotics is limited, and evidence of their prevalence in environmental reservoirs remains minimal (Dingiswayo et al., 2024). The *K. michiganensis* strains displayed an even broader resistance profile, with up to seven AMR genes. The commonly detected genes included *aph(3’)-Ia*, *bla*_OXY-1_, *fosA*, *mdf(A)*, *oqxAB*, and *tet(34)*, suggesting a conserved resistome across *Klebsiella* strains (Kang et al., 2020; Zhang et al., 2022). The *K. michiganensis* strain KM23 lacked only the *aph(3’)-Ia* gene. These findings align with earlier studies that identified *K. michiganensis* as an emerging multidrug-resistant opportunistic pathogen in clinical and environmental settings (Simoni et al., 2023; Long et al., 2024). The consistent detection of *fosA*, *oqxAB*, and *mdf(A)* genes in *Klebsiella* strains from livestock-associated water environments suggests persistent selective pressure likely driven by antibiotic runoff and manure contamination (Kang et al., 2020). These findings reinforce the role of such environments as reservoirs of transferable AMR genes and underscore the importance of genomic surveillance within a “One Health” framework (Zalewska et al., 2021). The virulence gene profiles of *K. variicola* and *K. michiganensis* strains isolated from a river near livestock farming areas reveal their strong environmental adaptability and potential to cause disease. *K. variicola* strains KvMW3 and KvMW4 carried 32 virulence genes, including iron-acquisition systems like *ent* and *fep* genes, fimbrial adhesion genes (*fimA-I*), and key outer membrane components such as *ompA* and *lpxC*. These features are essential for helping bacteria secure nutrients, adhere to surfaces, and evade the host immune response (Paczosa and Mecsas, 2016; Holt et al., 2015).

The *K. michiganensis* strains KMW6 and KMW7 had more diverse virulence profiles, with 36 genes identified. These included stress response genes (*katA* and *htpB*), magnesium transporters (*mgtB* and *mgtC*), and components of the type II secretion system (*gspE* and *gspG*), suggesting that these strains are metabolically flexible and capable of surviving harsh environmental conditions (Martin and Bachman, 2018). The *K. michiganensis* strains KM20 and KM26 were even more enriched, carrying 42 virulence genes, including *hcp-2*, *pla*, and *vipB/mglB*, which are linked to bacterial competition and increased toxicity (Yamada et al., 2024). The *K. michiganensis* strain KM23 had a similar profile but lacked *htpB* and *manB*, suggesting some differences in stress tolerance and capsule production. Across all *Klebsiella* strains, 29 virulence genes were consistently present. Antibiotic resistance genes like *ent*, *fep*, *fim*, *gmhA/lpcA*, *iutA*, *luxS*, and *ompA* were dominant, supporting bacterial colonization, biofilm development, and survival in low-nutrient environments (Follador et al., 2016). On the other hand, motility-related genes (*fle*, *fli motA* and *motB*) were rarely detected, which aligns with the generally non-motile nature of *Klebsiella* species observed in both clinical and environmental isolates (Wyres et al., 2020; Podschun and Ullmann, 1998; Holt et al., 2015).

Combining these virulence traits with antimicrobial resistance genes reinforces the potential of these bacteria to act as zoonotic threats within livestock-associated water systems (Zalewska et al., 2021). In these sequenced strains, virulence gene profiles revealed notable similarities between *K. variicola* and *K. michiganensis*. Several adhesive genes (*yagV/ecpE, yagW/ecpD, yagX/ecpC, yagY/ecpB*, and *yagK/ecpR)* and the iron-absorption gene (*fepC*) were present in both *K. variicola* and *K. michiganensis*. These adhesive genes are critical for colonization and infection, as they assist bacteria in adhering to host tissues and surfaces (Martin and Bachman, 2018). The iron-absorption genes, essential for infection in iron-limited environments, were absent in *K. michiganensis* strains (Chen et al., 2020; Kumar et al., 2024). The *mgtB* gene, which encodes a magnesium transporter and supports bacterial survival in low-magnesium environments and stress responses, was found only in *K. michiganensis* (Liu et al., 2024).

Comparative genomics demonstrate that the *bla*_LEN_ gene is prevalent in 75.3% of *K. variicola* strains (Lekota et al., 2024; Yang et al., 2024). In contrast, the *bla*_OX-1-3_ β-lactamase gene was discovered in *K. michiganensis* strains, marking the first report of *bla*_OX-1–3_ in *K. michiganensis* from a water stream in South Africa. However, the *bla*_OX-181_ carbapenemase-producing *K. michiganensis* isolate from hospital effluent in South Africa has been previously reported (King et al., 2021). The *aph(3’)-Ia_1* gene that confers resistance to streptomycin, which is one of the aminoglycoside resistances, was found exclusively in the *K. michiganensis* strain in this study and had been previously identified in South African hospital effluent (King et al., 2021). Both *K. pneumoniae, K. variicola* and *K. michiganensis* sequenced genomes contained the nalidixic acid resistance gene *oqxAB*, which encodes a multidrug efflux pump. This gene is found in the clinical groups of *Enterobacteriaceae* and plays a vital role in the resistance of bacteria to antibiotics and antimicrobial agents, including detergents and disinfectants (Li et al., 2019). The presence of these genes highlights the role of environmental water sources as reservoirs of antibiotic-resistant bacteria (Gomi and Adachi, 2025). These findings raise public health concerns and underscore the importance of genetic antibiotic testing, particularly for *K. michiganensis* and *K. variicola*, which are underreported and understudied from a “One Health” perspective in South Africa (Simoni et al., 2023).

Plasmid replicon analysis of *Klebsiella* genomes showed a diverse distribution of plasmid types, highlighting their critical role in spreading antimicrobial resistance. These *IncF*-replicon types are well recognized for carrying key resistance genes, such as *bla*_KPC_ and *bla*_NDM_, and for promoting horizontal gene transfer in clinical and environmental settings (Carattoli, 2009; Bi et al., 2018). In *K. michiganensis* strains KM20 and KM26, the presence of *IncFIB(pKPHS1_1_pKPHS1)* and *IncFII(Yp)_1* replicons suggest they share plasmid structures commonly associated with multidrug-resistant *Klebsiella* strains (Zhang et al., 2023). Interestingly, no identifiable replicon types were detected in *K. variicola* and *K. michiganensis* strains KMW6, KMW7, and KM23. A key limitation of this study is that several genomes particularly *K. variicola* isolates were untypeable using standard multilocus sequence typing (MLST) schemes due to missing loci in their assemblies. This incomplete representation of allele profiles may have reduced the accuracy of sequence-type assignment and potentially limited comparative genomic analyses.

### 4.1 Limitations

A key limitation of this study is that sequence types could not be assigned to some genomes due to missing MLST alleles, particularly among *K. variicola* strains. This incompleteness may reflect limitations inherent to short-read sequencing and draft genome assemblies, where fragmented contigs can lead to missing allele recovery. Alternatively, it is also possible that certain *Klebsiella* genomes genuinely lack these alleles due to genomic variation within the species. Consequently, this may have affected the resolution of population structure and comparative analysis. Future studies may incorporate long-read sequencing technologies to generate more complete assemblies, which could help clarify whether the missing alleles are due to technical constraints or represent true biological absence in these strains.

## 5 Conclusion

This study offers critical insights into the genetic and antimicrobial resistance profiles of *Klebsiella* species isolated from animal and environmental sources within a communal farming setting in the North West Province, South Africa. Detecting multidrug-resistant *K. pneumoniae*, *K. variicola*, and *K. michiganensis* strains, some of which carry antimicrobial resistance genes, underscores the complex and dynamic nature of bacterial populations at the human-animal-environment interface. High-risk clones such as mobile genetic elements like the AB595 plasmid in faecal isolates suggest potential zoonotic and environmental transmission pathways in shared-resource communities. These findings underscore the urgent need for integrated “One Health” approaches in rural South African communities to monitor, prevent, and mitigate the emergence and spread of antibiotic-resistant and potentially pathogenic *Klebsiella* species. Public health interventions must include regular environmental and veterinary microbiological assessments, improved sanitation infrastructure, and stewardship programmes o target antimicrobial use in livestock and protect both animal and human health in communal agricultural ecosystems.

## Data Availability

The datasets presented in this study can be found in online repositories. The names of the repository/repositories and accession number(s) can be found in the article/**Supplementary Material**.
