# Supplementary material for: Genomic characterisation and antimicrobial resistance of Klebsiella species isolated from small stock and environmental sources in South Africa
Source: Front Cell Infect Microbiol. 2026 Mar 11;16:1743913. doi: 10.3389/fcimb.2026.1743913 (PMC13013407; doi:10.3389/fcimb.2026.1743913)
Supplement: Supplementary file 1 [file Table1.docx]

**Supplementary**

**Genomic characterisation and antimicrobial resistance of *Klebsiella* species isolated from small stock and environmental sources in South Africa**

**Tshepang Motlhaping^1^,** **Deidre van Wyk^1^, Oriel Thekisoe^1^, Henriette van Heerden^2^, Kgaugelo E. Lekota^1*^, Tsepo Ramatla^3^**

^1^ Unit for Environmental Sciences and Management, North-West University, Potchefstroom 2520, South Africa.

^2^ Department of Veterinary Tropical Diseases, Faculty of Veterinary Science, University of Pretoria, Onderstepoort, 0110, South Africa.

^3^ Centre for Applied Food Safety and Biotechnology, Department of Life Sciences, Central University of Technology, 1 Park Road, Bloemfontein, 9300, South Africa.

***Correspondence:** Kgaugelo Lekota (PhD)

**Email Address:** lekota.lekota@nwu.ac.za


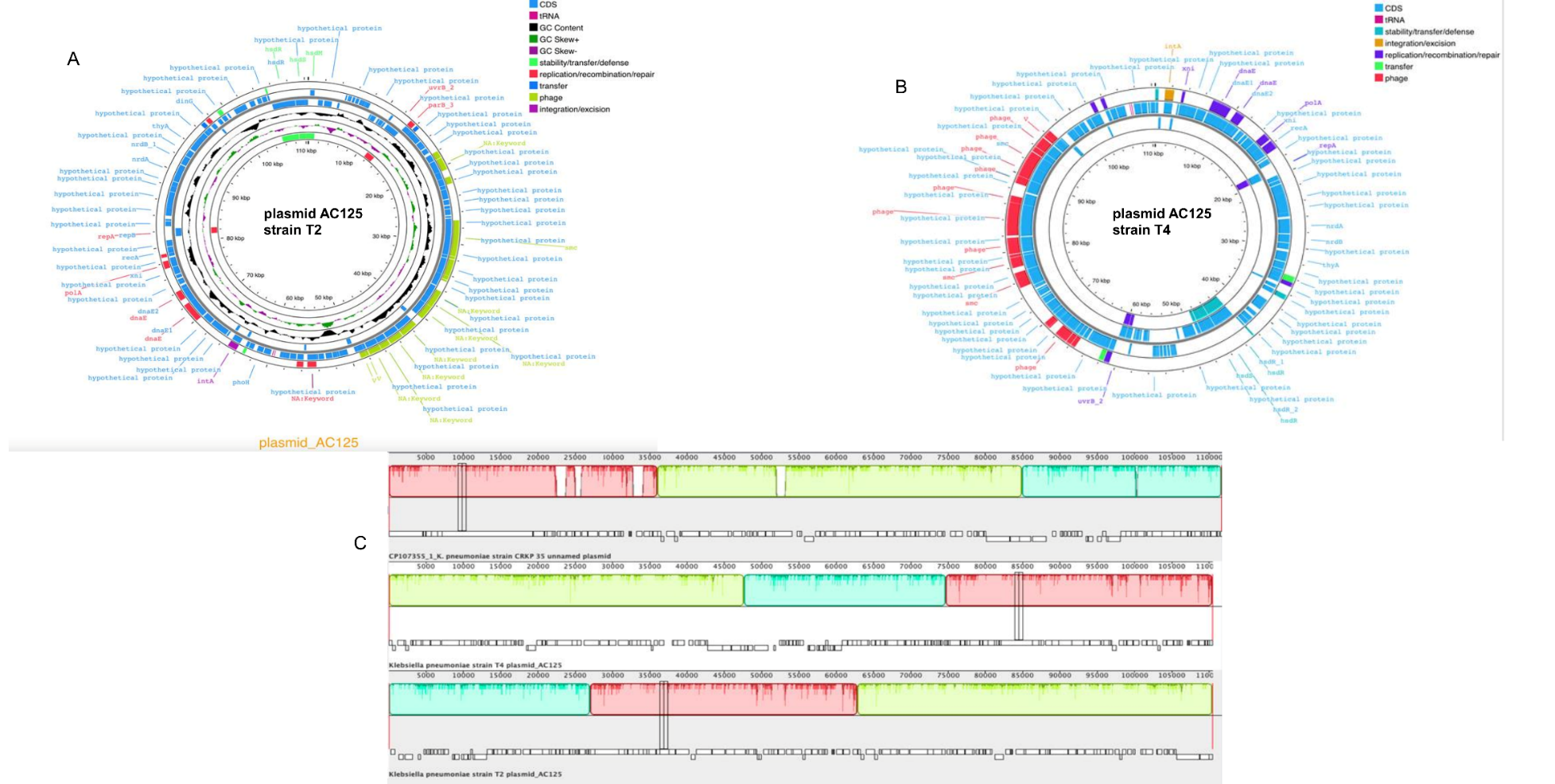


**Supplementary Figure S1:** Circular plots of plasmid AC125 identified in (A): *K. pneumoniae* strain T2. (B) and *K. pneumoniae* strain T4 (C). Multiple sequence alignment of *K. pneumoniae* CRkp-35 unnamed plasmid with plasmid AC125 identified in two *K. pneumoniae* strains. LCBs with the same colour indicate the nucleotide similarly, while LCBs with white blocks show differences of *bla*_KPC_ present in strain CRkp-35 and absent on the two plasmids AC125 sequenced in this study.


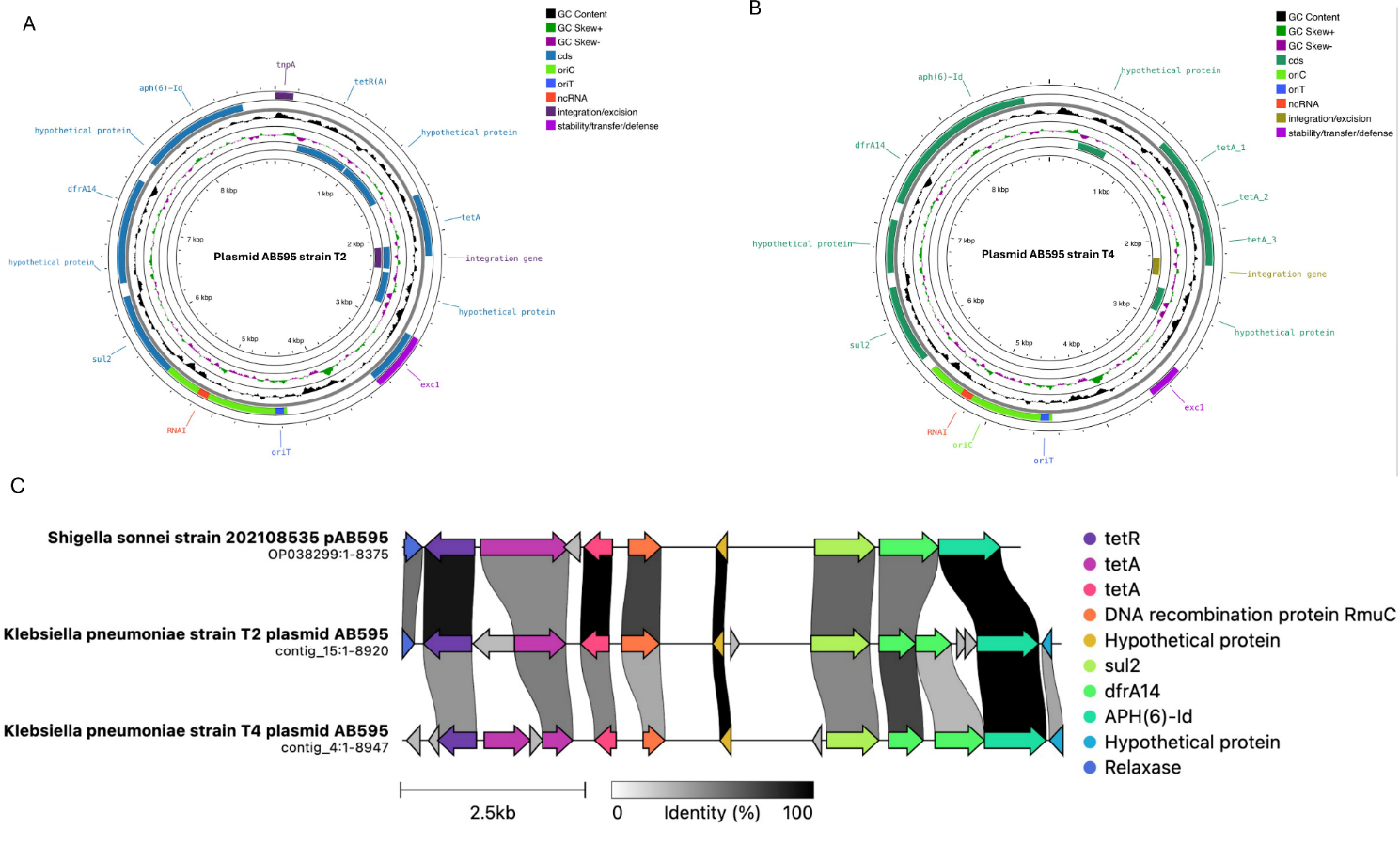


**Supplementary Figure S2:** (A) Circular plots of plasmid AB595 identified in *Klebsiella pneumoniae* strain T2. (B): Circular plot of plasmid AB595 identified in *K. pneumoniae* strain T4. (C): Annotation of the plasmids is based on the Bakta tool. Multiple sequence alignment of the plasmid AB595 sequenced in *K. pneumoniae* strains T2 and T4 in comparison with the *Shigella sonnei* strain 202108535 plasmid pAB585


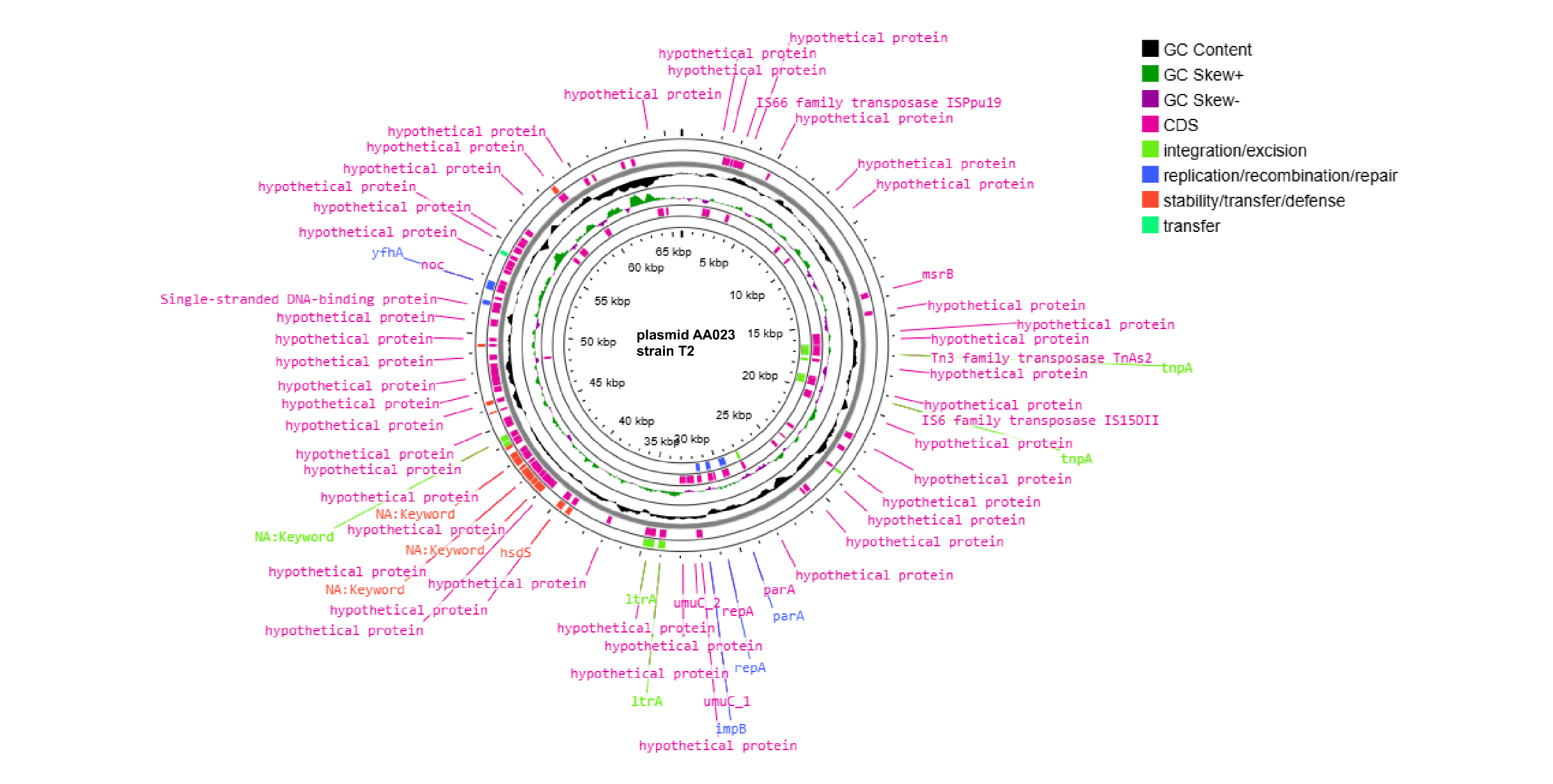


**Supplementary Figure S3:** Circular plot of plasmid AA023 identified in *K. pneumoniae* strain T2.

**Supplementary Table S1:** Genomes of the 84 *Klebsiella pneumoniae* strains and two sequenced strains used for comparative analysis retrieved from PATRIC and GenBank.

| **Genomes** | **Country** | **Hosts** | **Accession numbers** |
| --- | --- | --- | --- |
| 28spn | Sudan | Human | CP092527 |
| 39 | China | Human | JAQASO000000000 |
| 56spn | Sudan | Human | CP092528 |
| CNR146C9 | France | Human | OV408202 |
| DAFUVP011 | Switzerland | Human | DAFUVP011 |
| DAJMOO011 | Finland | Human | DAJMOO011 |
| E28 | South Africa | Human | JADIWQ000000000 |
| ECO3029993 | South Africa | Human | JADKPK000000000 |
| EDO1500733 | South Africa | Human/faeces | POWS00000000 |
| EFN299 | Ghana | Human | CP092589 |
| EGKP3 | Egypt | Human | CP046645 |
| EGKP4 | Egypt | Human | CP048801 |
| EGKP5 | Egypt | Cow | JABFHD000000000 |
| EGKP6 | Egypt | Cow | CP053359 |
| EGKP7 | Egypt | Chickens | CP048778 |
| HUM7199 | Botswana | Human | CP093314 |
| IMT44613 | Germany | Horses | CP104766 |
| ISO00199 | Ghana | Human | CP095140 |
| K35 | China | Human | JAQASS000000000 |
| KP167 | China | Environment | JAOAMR000000000 |
| KP168 | China | Environment | JAOAMQ000000000 |
| KP174 | China | Environment | JAOAML000000000 |
| KP176 | China | Environment | JAOAMJ000000000 |
| KP177 | China | Environment | JAOAMI000000000 |
| KP179 | China | Environment | JAOAMG000000000 |
| KP181 | China | Environment/Water | JAOAME000000000 |
| KP195 | China | Environment/ Water | JAOALU000000000 |
| KP196 | China | Environment/ Water | JAOALT000000000 |
| KP197 | China | Environment/ Water | JAOALS000000000 |
| KP202 | China | Environment/Water | JAOALN000000000 |
| KPI80 | South Africa | Human | SCLN00000000 |
| MI01K | India | Goat | CP084505 |
| MIN109 | Netherlands | Human | CP086128 |
| MM02K | India | Goat | JAHLYD000000000 |
| PN030E4 | Cameroon | Pig | PDVM00000000 |
| R307 | France | Human | CP114785 |
| SB612 | Netherlands | Environment/Water | CP084830 |
| SB615 | Netherlands | Environment/Water | CP084829 |
| SPC02401 | Sudan | Human | CP092807 |
| SPN0360 | Sudan | Human | CP092695 |
| SPN04301 | Sudan | Human | CP092697 |
| SPN04401 | Sudan | Human | CP092693 |
| SPN04701 | Sudan | Human | CP092694 |
| ST1552K086 | South Africa | Human | NXJP00000000 |
| ST2017950117510 | South Africa | Human | LJEF00000000 |
| TEMBI 15 | South Africa | Human | JANIUU000000000 |
| TEMBI19 | South Africa | Human | JANIUQ000000000 |
| TEMBI 2 | South Africa | Human | JANIUVF000000000 |
| TEMBI 21 | South Africa | Human | JANIUO000000000 |
| TEMBI 38 | South Africa | Human | JANIUD000000000 |
| TEMBI 39 | South Africa | Human | JANIUC000000000 |
| TEMBI 40 | South Africa | Human | JANIUB000000000 |
| Strain BIC1 | France | Human | CP022573 |
| Strain CFSAN054 | Denmark | Human | CP028180 |
| Strain D1 | France | Human | CP043969 |
| Strain SB5881 | France | Human | LR792628 |
| H8860 | South Africa | Human | VWTF00000000 |
| 6 | China | Human | JAQARR000000000 |
| CVUAS11890 | Germany | Sheep | PUIM00000000 |
| DT12 | Germany | Human | CP019079 |
| Iso00073 | Ghana | Human | CP095150 |
| Iso00262 | Ghana | Human | CP095132 |
| KP169 | China | Environment | JAOAMP000000000 |
| KZ00369997 | South Africa | Human | QMBX00000000 |
| MPUS7 | Tanzania | Human | CP047604 |
| PN085E1IA | Cameroon | Pig | PDVD00000000 |
| PN089E1 | Cameroon | Pig | PDVC00000000 |
| PR042E3 | Cameroon | Pig | PDVE00000000 |
| SB611 | Netherlands | Environment/Water | CP084843 |
| SB617 | Netherlands | Environment/ Water | CP084827 |
| SPC01301 | Sudan | Human | CP092805 |
| SPC03701 | Sudan | Human | CP092812 |
| SPN03101 | Sudan | Human | CP092809 |
| SPN01201 | Sudan | Human | CP092811 |
| TEMBI 8 | South Africa | Human | JANIVB000000000 |
| TEMBI 14 | South Africa | Human | JANIUV000000000 |
| TEMBI 17 | South Africa | Human | JANIUS000000000 |
| TEMBI 27 | South Africa | Human | JANIUL000000000 |
| TEMBI 29 | South Africa | Human | JANIUJ000000000 |
| TEMBI 31 | South Africa | Human | JANIUH000000000 |
| TEMBI 33 | South Africa | Human | [JANIUF000000000](http://www.ncbi.nlm.nih.gov/nuccore/JANIUF000000000) |
| Strain CNR48 | France | Human | LS399318 |
| Strain DT1 | Germany | Human | CP019079 |
| Strainkpn2166 | France | Human | [LR745045, LR745047, LR745046](http://www.ncbi.nlm.nih.gov/nuccore/LR745045,LR745047,LR745046) |
| T2* | South Africa | Sheep | JBIPSE000000000 |
| T4* | South Africa | Sheep | JBIPSD000000000 |

*Sequenced *K. pneumoniae* strains in this study.
